# Efficacy and safety of Yukgunja-Tang for treating anorexia in patients with cancer: The protocol for a pilot, randomized, controlled trial

**DOI:** 10.1097/MD.0000000000016950

**Published:** 2019-10-04

**Authors:** Hwi-Joong Kang, Mi-Kyung Jeong, So-Jung Park, Hyeong-Joon Jun, Hwa-Seung Yoo

**Affiliations:** a East-West Cancer Center, Dunsan Korean Medicine Hospital of Daejeon University; b Clinical Medicine Division, Korea Institute of Oriental Medicine, Daejeon, Korea.

**Keywords:** anorexia, cancer, clinical trials, Liu-Jun-Zi-Tang, malnutrition, Rikkunshito, Yukgunja-Tang

## Abstract

**Background:**

Anorexia is a common cause of malnutrition and is associated with negative effects on the quality of life (QOL) for patients with cancer. Management of appetite is the key to improving both the QOL and the prognosis for such patients. Yukgunja-tang (YGJT) is a traditional herbal medicine extensively prescribed in Korea as a remedy for various gastrointestinal syndromes. Currently, no standardized herbal medicine treatment exists for patients with cancer who are suffering from anorexia after surgery, chemotherapy, and/or radiotherapy. For that reason, this study aims to examine the efficacy and the safety of using YGJT to treat anorexia in such patients and to establish whether or not YGJT can be recommended as the primary therapy.

**Methods:**

We will enroll 52 cancer patients diagnosed with anorexia. The enrolled participants will be randomly allocated to 2 groups: The control group will receive nutrition counseling, and the YGJT group will receive nutrition counseling and be administered YGJT at a dose of 3 g twice a day for 4 weeks (a total of 56 doses of 3.0 g per dose). The primary outcome of this study is the change in the score on the anorexia/cachexia subscale (A/CS) of the Functional Assessment of Anorexia/Cachexia Therapy (FAACT). The secondary outcomes are the changes in the FAACT score with the A/CS score excluded, the score on the Visual Analogue Scale (VAS) for appetite, the weight and the body mass index (BMI), and laboratory tests for compounds such as leptin, tumor necrosis factor-α (TNF-α), ghrelin, and IL-6. All variables related to the safety assessment, such as vital signs, electrocardiography results, laboratory test results (CBC, chemistry, urine test), and adverse events, will be documented on the case report form (CRF) at every visit.

**Conclusion:**

This study is the first randomized controlled trial to investigate the efficacy and the safety of using YGJT for treating patients with cancer-related anorexia in Korea. We designed this study based on previous research about YGJT. This study will serve as a pilot and provide data for planning further clinical trials on herbal medicine and cancer-related anorexia.

**Trial registration:**

Clinical Research Information Service (CRIS), Republic of Korea, ID: KCT0002847. Registered retrospectively on 3 April 2018.

## 1 Introduction

Anorexia is defined as an involuntary loss of appetite.^[1]^ It is a common cause of malnutrition and is associated with negative effects on the quality of life (QOL) in patients with cancer.^[2,3]^ Its symptoms involve decreased dietary intake and body weight loss if it gets worse. Studies have shown that 30% to 60% of patients with cancer-related anorexia are malnourished, which is usually associated with long hospitalization.^[4,5]^ Thus, one may conclude that cancer and its treatments may cause malnutrition and side effects that affect the nutritional status of patients suffering from the condition.^[3]^

Weight loss caused by malnutrition is associated with poor prognosis, such as short median survival, low chemotherapy response rate, and decreased performance status.^[6]^ Therefore, cancer-related anorexia, which is usually followed by loss of weight, is a distressing symptom for patients with cancer. This symptom may progress to precachexia, cachexia, and sarcopenia. Relieving anorexia has not only a supplementary, but also an essential therapeutic, aim of improving both the QOL and the prognosis for such patients.

Radical treatment to relieve cancer-related anorexia and cachexia syndrome (CACS) is assuredly required if patients are to be cured. However, if such an approach does not work, another option would be to increase nutritional intake through dietary counseling and education and through the administration of oral nutritional supplements. Appetite stimulants, such as corticosteroids (dexamethasone, methylprednisolone, and prednisolone) and progestins (medroxyprogesterone and megestrol acetate) are recommended for patients with anorexia.^[7]^ However, long-term and high-dose use of corticosteroids has been found to be associated with significant adverse events, including immunosuppression, hyperglycemia, muscle weakness/wasting, and fluid retention.^[8]^ Adverse events associated with the use of progestins include peripheral edema, hyperglycemia, hypertension, thrombotic events, breakthrough vaginal bleeding, Cushing syndrome, alopecia, and adrenal suppression, leading to adrenal insufficiency if abruptly stopped.^[9]^ The 2 above-mentioned categories of drugs, which are recommended as standard therapy for patients suffering from CACS, have been used for patients in whom short-term benefit is desired and for those with months-to-weeks life expectancy.^[8,10]^

Some patients seek to receive alternative safe and effective therapies such as herbal medicines for the treatment of cancer-related anorexia.^[11]^ Yukgunja-tang (YGJT), also known as Rikkunshito in Japan and Liu-Jun-Zi-Tang in China, is a traditional herbal medicine extensively prescribed in East Asian countries for the treatment of patients with various gastrointestinal syndromes, such as anorexia, nausea, dyspepsia, gastritis, and gastroesophageal reflux disease.^[12–18]^ A number of experimental studies have shown that YGJT may be effective in treating cisplatin-induced anorexia.^[19–21]^ However, additional clinical evidence for YGJT being a practical approach to treating such patients is needed. Therefore, we designed a randomized, controlled clinical trial to examine the efficacy and the safety of using YGJT to treat anorexia in patients suffering from cancer. Also, this pilot study will be performed to gather more evidence in support of recommending YGJT as a standard treatment for patients with cancer-related anorexia.

## 2 Methods

### 2.1 Study design

**Figure 1:**
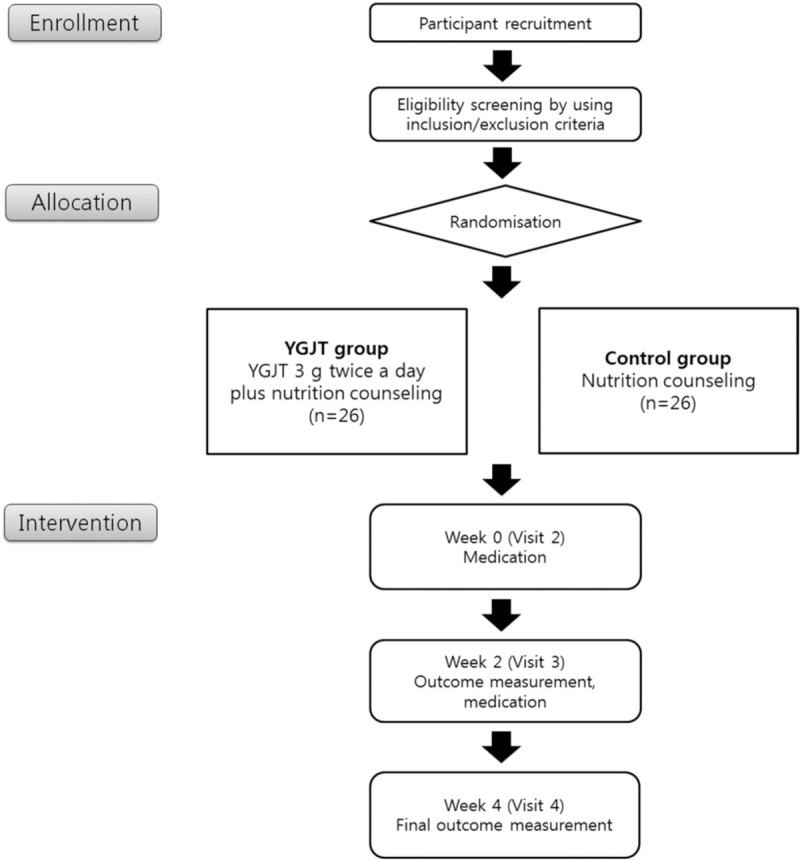
Study flow chart of the YGJT clinical trial.

This study is a double-blind, randomized, controlled trial that aims to examine the efficacy and the safety of using YGJT to treat patients with cancer-related anorexia. All patients fulfilling the eligibility criteria will be selected. The enrolled participants will be randomly allocated to 2 groups: The control group will receive nutrition counseling, and the YGJT group will receive nutrition counseling and be administered YGJT. Figure 1 shows the schematic flow of this YGJT clinical trial.

### 2.2 Recruitment

Participants will be recruited through advertisement on bulletin boards at and the homepage of Dunsan Korean Medicine Hospital of Daejeon University in Daejeon, Republic of Korea. Detailed information on the trial, including study period, purpose of study, inclusion and exclusion criteria, and intervention, will be posted on a recruitment notice, and those patients who sign the informed consent forms will voluntarily visit the trial site.

### 2.3 Inclusion criteria

Participants meeting the following criteria will be included: Patients who have a histologically or cytologically confirmed solid tumor; individuals who are suffering from anorexia; men and women aged 20 to 80 years who can write, read, and understand; score on the Visual Analog Scale (VAS) for anorexia ≥ 40/100 mm; neutrophils ≥ 1500/μL, platelets ≥ 100,000/μL; total bilirubin of the maximum normal level or less (1.2 mg/dL); alanine aminotransferase (ALT) and aspartate aminotransferase (AST) levels lower than twice the upper limit for normal (35/45U/ℓ); creatinine level lower than 1.5 times the upper limit for normal (1.09 mg/dL); patients who provide written informed consent for participation in the trial.

### 2.4 Exclusion criteria

Participants meeting one or more of the following criteria will be excluded: Patients unable to intake medicine orally; patients who are undergoing chemotherapy and those who completed such therapy less than 1 month earlier; patients who have survived at least 5 years after having been diagnosed with cancer; Eastern Cooperative Oncology Group (ECOG) performance status score ≥ 3; patients who have dementia, delirium or depression; patients who score more than 7 points on the Numeric Pain Rating Scale within 2 weeks of screening; patients who have diseases like hypoadrenalism that can influence appetite; patients who are taking appetite stimulants such as megestrol acetate, corticosteroids, and thalidomide; patients who are judged to be inappropriate for inclusion in this study; women who are pregnant or are planning to become pregnant.

### 2.5 Withdrawal criteria

Participants who meet the withdrawal criteria will be dropped from the trial. Participants who withdraw or are dropped after randomization will be followed for outcomes. The withdrawal criteria include the use of any forbidden medication or treatment during the trial that could affect the study result, the participant's withdrawal of consent, the occurrence of a serious adverse event, the detection of eligibility violations, the occurrence of other significant protocol violations during the trial, and the investigator's decision to terminate the process for the sake of the participant's health.

### 2.6 Sample size

This study is an exploratory trial conducted before a confirmatory trial to establish the validity of using YGJT to treat patients with cancer who are suffering from anorexia. The efficacy of YGJT will be examined in order to provide information that will lead to a better design for a future clinical trial. Considering a dropout rate of 20%, we calculated that a total of 52 subjects, 26 per group, should be enrolled in this study. The sample size was set using an effective size of 0.9, which is the ratio of the standard deviation of each group from the mean difference of outcomes between the YGJT group and the control group at a significance level of 5% and a power of 80%. The formula for estimating the sample size is as follows:  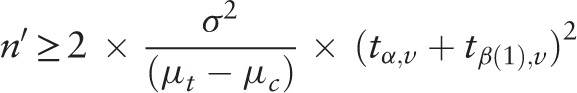



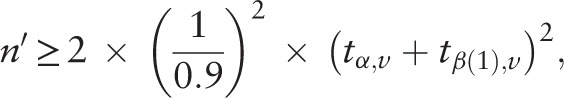


where n is the approximate sample sizes required per group, *t*_*α,v*_ is the 97.5 percentile score of the *t* distribution with *v* degrees of freedom, and *t*_*β*(1),*v*_ is the 80 percentile score of the *t* distribution with *v* degrees of freedom. If *v* = 40, n′ ≥ 2 × 1.2346 × (2.0211 + 0.8507)^2^ = 20.3632 (21). For a dropout rate of 20%, the sample size for each group is given by n = 21 × 1.2 = 25.2 (26). Therefore, a total of 52 subjects, 26 in each of the 2 groups, will be enrolled in this study.

### 2.7 Randomization and allocation

After informed consent has been obtained, random assignment will be performed by using a computer-generated, blocked, random-allocation sequence with an allocation ratio of 1:1. Each participant will be assigned to either the YGJT group or the control group according to a pregenerated randomization table and an assigned randomized number, which will be prepared in advance. Computer-generated block randomization, with block sizes of 4 and 8, will be done by a member of the Department of Information and Statistics, Chungnam National University, Daejeon, Korea; that member will have no clinical involvement in the trial. This procedure will ensure that the allocation is securely concealed from the researchers who will subsequently inform the patients of their allocations.

### 2.8 Interventions

The participants in the control group will be provided nutrition counseling on recommended chemotherapy, radiation, postoperative, and cancer-preventive diets. In addition to nutrition counseling, participants in the YGJT group will be administered YGJT at a dose of 3 g twice a day before meals for 4 weeks, for a total of 56 doses of 3.0 g per dose. Kolmarpharma Co, Ltd, produces the YGJT in accordance with Korea Good Manufacturing Practice (KGMP) standards. YGJT is manufactured by spray-drying a hot water extract of a mixture of 8 varieties of the following crude drugs: Atractylodis lanceae rhizoma (4.0 g), Ginseng radix (4.0 g), Pinelliae tuber (4.0 g), Poria cocos (4.0 g), Zizyphi fructus (2.0 g), Aurantii nobilis pericarpium (2.0 g), Glycyrrhizae radix (1.0 g), and Zingiberis rhizoma (0.5 g). These raw materials will be extracted and concentrated to 3 g per dose. The daily doses follow the recommended dosage method of the Korea Ministry of Food and Drug Safety (MFDS). During the trial, participants will be prohibited from receiving any other treatment for anorexia.

### 2.9 Outcome measurement

#### 2.9.1 Primary outcomes

The primary outcome is the change in the score on the anorexia/cachexia subscale (A/CS) of the Functional Assessment of Anorexia/Cachexia Therapy (FAACT) between baseline (visit 2) and the end of the administration period (visit 4). The FAACT is a clinical questionnaire for assessing the general aspects of QOL, as well as specific anorexia-related concerns, in patients with cancer. Especially, the A/CS has 12 items focusing on anorexia and cachexia among the total 39 items. The Korean version of FAACT, which was translated by the Functional Assessment of Chronic Illness Therapy (FACIT) organization, will be used. A trained investigator using standards operating procedures (SOPs) will determine and record the A/CS score at each visit.

#### 2.9.2 Secondary outcomes

Secondary outcomes are the changes in the FAACT score with the A/CS score omitted, in the VAS scorer for appetite, in the weight and the body mass index (BMI), and in the levels of leptin, tumor necrosis factor-α (TNF-α), ghrelin, and IL-6. The study schedule is summarized in Table 1.

**Table 1:**
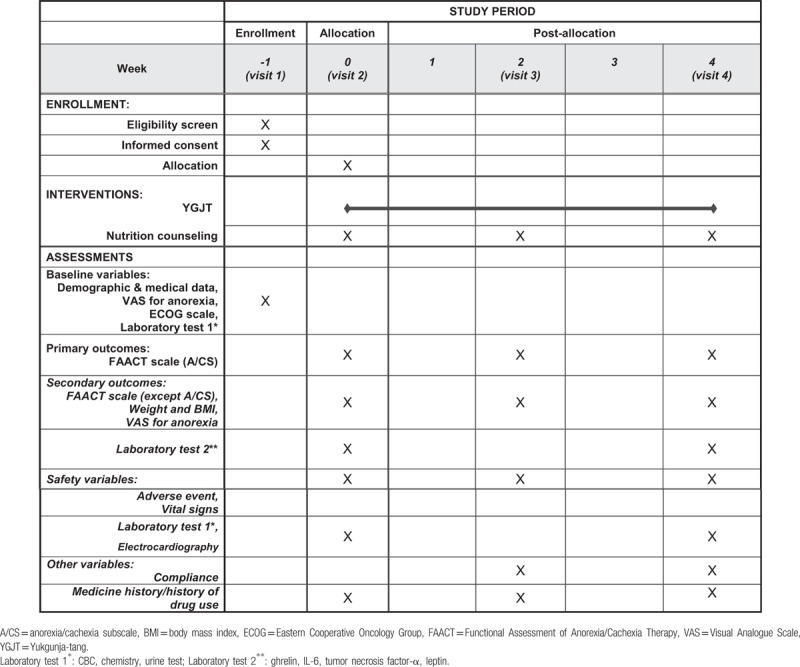
Summary the timetable for the YGJT clinical trial.

#### 2.9.3 Safety outcomes

All variables related to the safety assessment, such as vital signs; electrocardiography, CBC, chemistry, and urine test results; and adverse events, will be documented on the case report form (CRF) at every visit.

#### 2.9.4 Subgroup analysis

Participants will be subdivided into the following 3 subgroups: patients who complain of anorexia after surgery, patients who complain of anorexia during radiation therapy or recent radiation therapy, patients who complain of anorexia more than 1 month after chemotherapy. Comparisons of the YGJT group and the control group will also be performed at the subgroup level.

### 2.10 Statistical analysis

#### 2.10.1 Efficacy assessment

The Shapiro–Wilk test will be used to test normality for the mean changes of primary and secondary outcomes between baseline (visit 2) and the end of treatment (visit 4). A significance level 0.05 will be used to test the differences between the 2 groups. The statistical significance of the variables in 2 groups will be tested by using either the paired *t* test or the Wilcoxon signed rank test. The present study has no plans for interim analysis.

#### 2.10.2 Safety assessment

Adverse events (AEs) will be reported and recorded in detail during the entire study. The following will be used to assess safety: vital signs, CBC, chemistry, and urine tests, and electrocardiography. Safety-related variables will be analyzed using the intention to treat (ITT) data set. Comparisons between the ratio of the number of participants who experienced 1 or more adverse events to the total number in that group or subgroup will be performed using a chi-square analysis or Fisher exact test. The statistical significance of the changes between subgroups/groups will be tested using the 2-sample *t* test or the Mann–Whitney test. In addition, the statistical significance of changes within each group will be tested using the paired *t* test or Wilcoxon singed-rank test. Electrocardiography results and some urine-test results will be classified as normal and abnormal according to clinical significance, and comparisons within groups will be analyzed using the McNemar test.

#### 2.10.3 Subgroup analysis

The efficacy of YGJT between subgroups will be evaluated as follows: comparison of the YGJT efficacy for each of the subgroups in the YGJT group with that of the corresponding subgroup in the control group and comparison of the YGJT efficacy among the 3 subgroups of the YGJT group. The statistical significance of any differences between the YGJT group and the control group, among the 3 subgroups, will be analyzed using a generalized linear model with either the Wilcoxson rank sum test or the Kkruskal–Wallis test. All tests will be 2-sided tests, and the significance level will be 0.05.

### 2.11 Quality control

Regular monitoring will be conducted by checking trial master files, CRFs, informed consent forms, compliance with treatments, AEs, and data records to ensure the accuracy and quality. The management, data collection, analysis, and reporting of the study will be conducted independently by the study investigators.

### 2.12 Ethics approval and dissemination

The Institutional Review Board of Dunsan Korean Medicine Hospital of Daejeon University approved the study (reference DJDSKH-17-DR-17). The current protocol version is 2.2. The trial will be performed in compliance with the Helsinki Declaration and according to Good Clinical Practice as described by the Korea Food and Drug Administration.

The confidentiality of personal information will be protected during and after this clinical trial. Each participant will be assigned a study identification number at enrollment. Throughout the trial, data will be managed using the study identification number. Only investigators will have the right to access the data. The results of this clinical trial will be disseminated through publications in scientific journals and presentations at scientific conferences.

## 3 Discussion

This pilot study will investigate the clinical efficacy and safety of using YGJT to treat patients with cancer-related anorexia. The QOL of patients is influenced by nutritional status.^[22,23]^ Thus, anorexia accompanied by reduced food intake is a significant issue in the management of such patients because it contributes to the development of malnutrition, increases morbidity and mortality, and has an adverse influence on QOL. Thus, the burden of anorexia can be alleviated by comprehensive nutrition care tailored to meet the needs of the patients as their physical conditions change. Nutritional counseling is the first and most commonly utilized intervention for the management of malnourished cancer patients and for the treatment of problems with the gastrointestinal tract.^[24]^ This pilot study will provide all participants with nutritional education and counseling^[25,26]^ by a professional dietitian.

Recently, several herbal medicines have been used for the treatment of patients suffering from anorexia,^[11,16,27]^ and the mechanisms have been reported.^[28–30]^ Several studies reported a correlation between YGJT and plasma ghrelin levels in cancer patients with chemotherapy-induced anorexia.^[21,31]^ YGJT stimulates ghrelin secretion from the stomach, and the response to it in the hypothalamus regulates plasma ghrelin levels, sensitizes ghrelin receptors, and antagonizes 5-HT2b/c receptors.^[19,32]^ We designed this study based on the results of previous research about YGJT.

This study is the first trial in Korea of YGJT focused on treating patients with cancer suffering from anorexia. Usually, herbal medicines such as YGJT have not been initially recommended as remedies for treating various symptoms because they consist of multiple components whose active substances, pharmacological functions, and multiple mechanisms have not been clearly elucidated. Consequently, additional mechanistic studies are needed in the future to determine definitely the effects of YGJT.

Even though this trial may provide scientific evidence for the efficacy of YGJT in relieving anorexia, this study has limitations that are mostly derived from its relatively small sample sizes. In addition, identifying clearly whether anorexia resulted from cancer or from cancer treatment, such as surgery, chemotherapy, or radiotherapy, is problematic. Therefore, the particular mechanisms underlying anorexia suffered by cancer patients cannot be clearly determined. Considering recruitment feasibility, patients with various cancer types and with different stages of cancer will be recruited in this pilot study. Further studies are needed to estimate the influences of both the specific types and stages of cancer and the surgical status. Despite these limitations, this study should serve as a pilot report to plan further clinical trials on herbal medicine and cancer-related anorexia.

## Author contributions

**Conceptualization:** Hwi-Joong Kang, Mi-Kyung Jeong.

**Investigation:** Hwi-Joong Kang, Mi-Kyung Jeong.

**Writing – original draft:** Hwi-Joong Kang, Mi-Kyung Jeong, So-Jung Park.

**Writing – review & editing:** Hwi-Joong Kang, Mi-Kyung Jeong, Hyeong-Joon Jun, Hwa-Seung Yoo.

Hwi-Joong Kang orcid: 0000-0002-6569-538X.
